# Lymphocyte Differentiation and Effector Functions

**DOI:** 10.1155/2012/510603

**Published:** 2012-12-03

**Authors:** Niels Olsen Saraiva Camara, Ana Paula Lepique, Alexandre S. Basso

**Affiliations:** ^1^Department of Immunology, Institute of Biomedical Sciences, University of São Paulo, 05508-000 São Paulo, SP, Brazil; ^2^Departamento de Microbiologia, Imunologia e Parasitologia, Escola Paulista de Medicina, Universidade Federal de São Paulo, 04021-001 São Paulo, SP, Brazil

Lymphocytes are essential in combating infections; they display powerful effector mechanisms and their activity must be regulated at all times to avoid self-tissue or cells destruction. These immunological lineages are detected in protochordates and vertebrates. Long-term hematopoietic cells originate, in the bone marrow, all hematopoietic lineages, including lymphocytes. B and T lymphocytes are responsible for adaptive immune responses. Natural killer cells, NK, are also considered a lymphocytic lineage; however, its development is completely different from that of lymphocytes. NK cells display a different set of receptors from those expressed by B and T lymphocytes and are responsible for innate, not adaptive responses against virus infected and tumor cells. Finally, NKT cells are also lymphocytes generated in the thymus through contact with glycolipid loaded CD1d-presenting cells with a diverse function in modulating immune responses against self- and foreign antigens.

Function of lymphocytes and its products range from the neutralization of pathogens with specific antibodies to the activation of macrophages and to direct cytotoxic activity. Lymphocytes differentiate in primary lymphoid organs where they commit a lymphocytic lineage, express B or T cell receptors (BCR and TCR, resp.,), which are essential for cell survival and further maturation as well as function, and are selected according to their capacity of antigen recognition. Virtually all antigens present or presented in primary lymphoid organs are self-antigens. Lymphocytes that express receptors with high affinity to self-antigens either trigger programmed cell death or differentiate into regulatory cells (natural regulatory T cells). Lymphocytes that succeed in expressing a functional receptor with low-to-moderate affinity to self-antigens emigrate to secondary lymphoid organs, where they are exposed to foreign antigens and may be activated to generate effector responses (Figure 1). While B cells develop, in mammals, in the bone marrow, T cell progenitors migrate to the thymus to develop to mature TCRalpha/beta CD4 and CD8 T cells, as well as TCRgamma/delta T cells. During development in primary lymphoid organs, lymphocytes depend on a series of signals to pass through the checkpoints necessary to generate mature cells. In all progenitor stages, interaction with the organ stroma is important, but soluble factors as cytokines are also important for the survival of progenitor cells, mainly before B or T receptor expression. Y. Wanget al.  review the expression control and role of Bcl-xL, a protein that promotes cell survival, in T cell development in the thymus as well as in T cell activation in the periphery.

An interesting feature of lymphocyte progenitors that migrate to the thymus is their potential to originate other lineages. Besides T cells, these progenitors have the potential to originate NK cells, dendritic cells, and B cells. Notch signalling is necessary for T cell fate determination. M. Braunstein and M. k. Anderson bring HEB (HeLa E box binding factor) to the spotlight in the review about its role in T cell commitment and transition through CD4^−^CD8^−^ stages of differentiation. Interestingly, HEB^−/−^ DN3 thymocytes can originate NK cells in the thymus. In the mouse embryo, mature NK cells are found in the thymus, but immature, therefore potential NK progenitors, are found in the bone marrow, spleen, and liver. X. Wu et al. compare the development of NK cells in the spleen and liver in the mouse embryo. Their data show that the expression of adhesion molecules as CD11c and CD73 in liver NK cells may account for the higher frequency of these cells in this tissue compared to others and that the liver microenvironment has a role in NK differentiation. Focusing on the cell membrane, instead of cytoplasmic and nuclear factors, as Bcl-xL and HEB, B. Jin et al. review the role of the Toll like receptors in T cell differentiation and activation. This review brings information on the effect of different TLR ligands on T cell development and the effect of activation of different TLRs in antigen presentation, tolerance control, and T cell activation. Regarding interaction with stroma of primary lymphoid organs, R. Romano et al. review the role of FOXN1 in T cell development and primary immunodeficiencies caused by its altered expression in stromal cells in the thymus. 

Once mature lymphocytes are generated, they migrate to secondary lymphoid organs where they may encounter antigens and depending on the conditions of this encounter, they may be activated to generate effector responses. Activation of B and T lymphocytes displays some common and some different aspects. Both must recognize the antigen through its B or T cell receptor. However, while BCR binds directly to the antigen, the T cell receptor only binds to antigen presented by antigen presenting cells through the Major Histocompatibility Complex (MHC). This binding takes place in a super structure called Immunological Synapse, where adhesion molecules, costimulatory molecules, and receptors, besides TCR and MHC, are present. Both types of lymphocytes need more than the antigen to mount an efficient effector response. For example, B cells may respond to ligands of TLR besides BCR, and T cells have receptors for costimulatory molecules presented by antigen presenting cells, which are expressed upon proinflammatory signals, as ligands for TLRs. Absence of a second stimulus besides activation of BCR or TCR promotes the induction of anergy or regulatory responses. Upon activation and depending on signals presented to lymphocytes during activation, these cells will differentiate into subtypes with specific functions. CD4 T cells undergo differentiation into CD4 helper phenotypes, as discussed in several articles in this special issue. Already primed B cells may encounter primed T cells and the communication between these two lineages in the lymph nodes will promote isotype switching, affinity maturation, and proliferation in B cells, as well as proliferation and further activation in T cells.

A. Visekruna et al. review the role of the transcription factor NF*κ*B in the T cell activation and effector functions. R. V. Luckheeram et al. discuss stimuli that promote CD4 T cell differentiation in the known T cell Th subtypes, activation, and plasticity and effector functions. With a very different approach, R. von Essen et al. discuss, in a broad review, the concept of avidity maturation in T lymphocytes and signals involved in such mechanism. As mentioned before, for activation lymphocytes need at least two signals, in this review, cytokines are considered the “third” signal to naive and primed T cell activation and differentiation. Cell polarity in different steps of T cell activation and differentiation is discussed in the review by I. Fung et al., with focus on GTPases, which are involved in cell migration in several immune lineages, and the DOCK8 protein a Rho-Rac guanine exchange factor. 

During lymphocyte activation, different stimuli will influence the differentiation of memory cells, which are important for the control of new infections by the same pathogen. M. N. Norazmiet al. discuss the expression and role of Peroxisome proliferator-activated receptor *γ* 1 and 2 (PPAR*γ*) in human naive and memory T cells upon TCR activation. Data presented by the authors suggest that the two PPAR*γ*  isoforms may have different roles during the activation of naive and memory T cells.

Research on lymphocyte biology has a strong bias towards clinical aspects and mechanism of several diseases, from cancer to graft transplantation rejection to pathogen immune responses. This special issue brings one article and one review regarding inhibition of allograft rejection. Antigen presentation and the role of B7 costimulatory molecule in allograft rejection are explored in the article by Y. F. Yao et al. Using an antisense B7 peptide, the authors were able to inhibit T cell alloactivation and inhibit arterial allograft intimal hyperplasia in a murine allogeneic carotid transplant model. R. Wang et al. discuss a population that has been actively studied in cancer, where it plays a protumoral role, but that may be beneficial for the survival of allotransplants. The myeloid derived suppressor cells are described and their potential role in inhibiting cardiac allograft.

C. Wickenhauser et al. describe different thresholds of the activation of B cells by antigen and hapten in patients with leukocyte adhesion-deficiency 1 (LAD1). Contrary to immunodeficiency, patients with chronic C hepatitis frequently develop thyroid disorders during IFN*α* therapy. Y. Kajiyama et al. show that female patients with a higher serum concentration of BAFF (B-cell-activating factor) display significantly a higher risk of developing B cell dependent thyroid disorders, as Graves disease and the production of thyroid auto antibodies.

Finally, C. Schlimperet al. related their experience in the generation of CIK (Cytokine Induced Killer cells) engineering lymphocytes from colorectal carcinoma patients, using the CAR chimera, chimeric antigen receptor, which binds to the carcinoembryogenic antigen. Their data indicate that this approach was successful in inducing patient T cell proliferation and IFN*γ* production in an antigen dependent manner.

This special issue covers several important aspects of lymphocyte development, differentiation, and function bringing relevant and up-to-date information in this area.

*Niels Olsen Saraiva Camara* *Ana Paula Lepique* *Alexandre S. Basso*

## Figures and Tables

**Figure 1:**
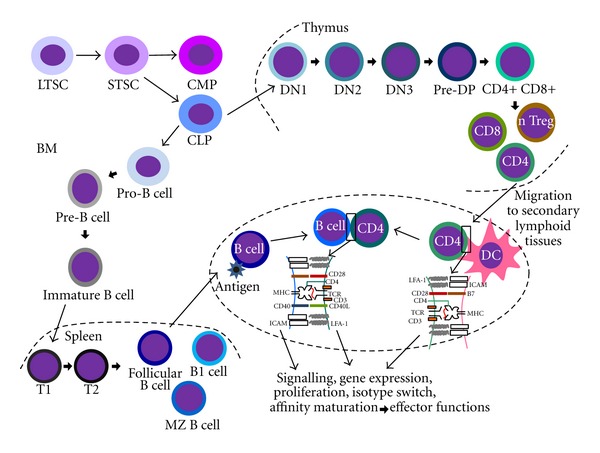
Schematic representation of lymphocyte development and activation. Development of lymphocytes takes place in primary lymphoid organs as the bone marrow (BM) and the thymus. Long-term hematopoietic stem cells (LTSCs) generate short-term hematopoietic stem cells (STSCs), which in turn generate common myeloid and lymphoid progenitors (CMP and CLP, resp.,). T cell progenitor, migrate to the thymus, where they undergo maturation through stages known as double negative (DN), representing CD4^−^CD8^−^ cells, double positive, and finally mature CD4^+^ or CD8^+^ T cells. Immature B cells leave the bone marrow to finish their development in the spleen, where they progress through transitional stages 1 and 2 (T1 and T2) to generate mantle-zone B cells (MZ B cells), follicular B cells, or B1 cells. All mature lymphocytes circulate through secondary lymphoid organs, where they are exposed to antigens, directly or through antigen presenting cells. After the first stimulation by antigens, B and T cells migrate towards each other to interact in a process that will determine B cell antibody production and T cell proliferation and further activation. The immunological synapses are represented between a dendritic cell and a naive T cell and between primed B and T cells. In detail, one may observe molecules present in immunological synapses.

